# Supplementary material for: SARS-CoV-2 seropositivity and COVID-19 among 5 years-old Amazonian children and their association with poverty and food insecurity
Source: PLoS Negl Trop Dis. 2022 Jul 18;16(7):e0010580. doi: 10.1371/journal.pntd.0010580 (PMC9292121; doi:10.1371/journal.pntd.0010580)
Supplement: S1 Fig — The map shows Brazil and Peru in South America (panel A) and the location of the municipality of Cruzeiro do Sul (dark green) in the Upper Juruá Valley region, Acre State (light green), northwestern Brazil (panel B). The urban area of Cruzeiro do Sul is shown in greater detail in panel C. Other cities and towns in the region (Mâncio Lima, Guajará, and Rodrigues Alves) are also indicated by triangles. Roads and streets are represented in light gray. Rivers are represented in blue. Figure created with QGIS software version 3.14, an open-source Geographic Information System (GIS) licensed under the GNU General Public License (https://bit.ly/2BSPB2F). Publicly available shapefiles were obtained from the Brazilian Institute of Geography and Statistics (IBGE) website (https://bit.ly/34gMq0S). Roads, streets, and rivers were obtained from the Open Street Map Foundation website (https://bit.ly/3pzh4xp). All utilized geographical data are under the Creative Commons Attribution License (CC BY 4.0). Reproduced from: Pincelli A, Cardoso MA, Malta MB, Johansen IC, Corder RM, Nicolete VC, et al. Low-level Plasmodium vivax exposure, maternal antibodies, and anemia in early childhood: Population-based birth cohort study in Amazonian Brazil. PLoS Negl Trop Dis. 2021;15:e0009568. doi: 10.1371/journal.pntd.0009568. (PDF) [file pntd.0010580.s002.pdf]

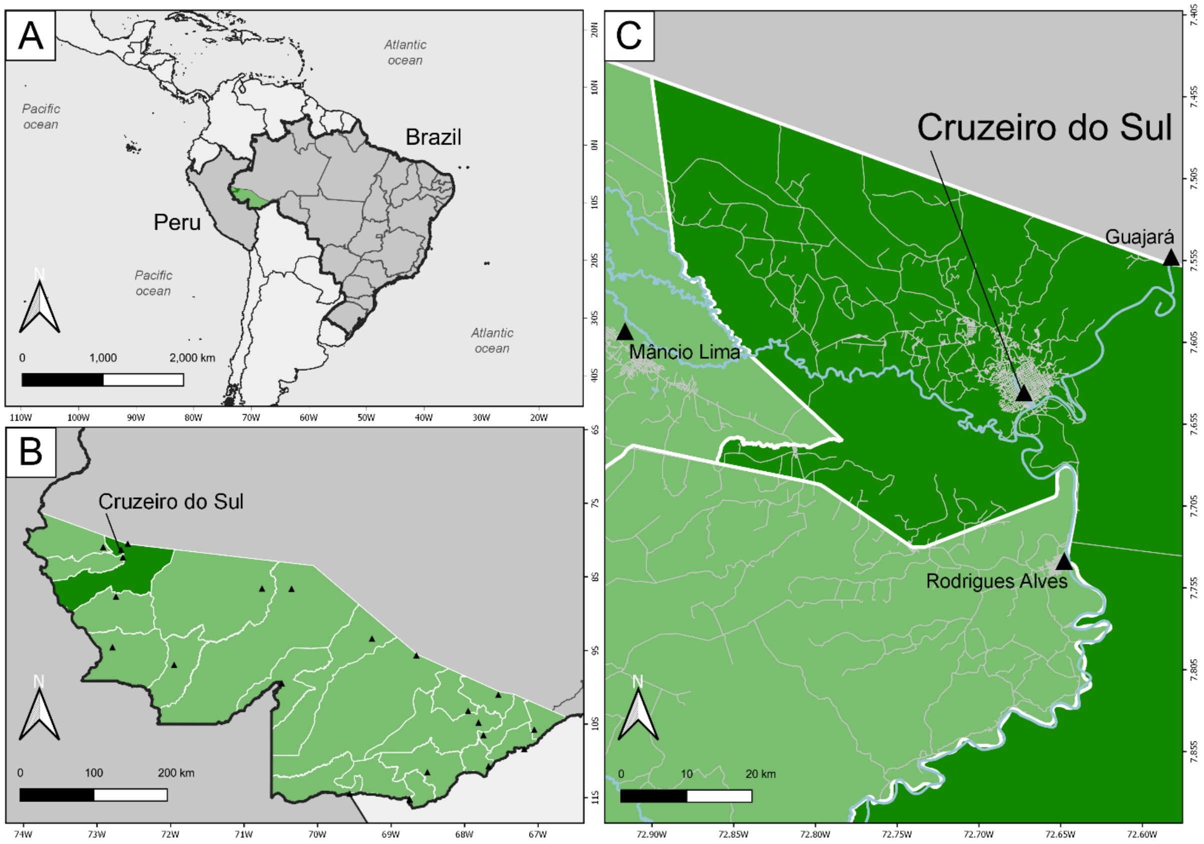

S1 Fig. Study site. The map shows Brazil and Peru in South America (panel A) and the location of the municipality of Cruzeiro do Sul (dark green) in the Upper Juruá Valley region, Acre State (light green), northwestern Brazil (panel B). The urban area of Cruzeiro do Sul is shown in greater detail in panel C. Other cities and towns in the region (Mâncio Lima, Guajará, and Rodrigues Alves) are also indicated by triangles. Roads and streets are represented in light gray. Rivers are represented in blue. Figure created with QGIS software version 3.14, an open-source Geographic Information System (GIS) licensed under the GNU General Public License (https://bit.ly/2BSPB2F). Publicly available shapefiles were obtained from the Brazilian Institute of Geography and Statistics (IBGE) website (https://bit.ly/34gMq0S). Roads, streets, and rivers were obtained from the Open Street Map Foundation website (https://bit.ly/3pzh4xp). All utilized geographical data are under the Creative Commons Attribution License (CC BY 4.0). Reproduced from: Pincelli A, Cardoso MA, Malta MB, Johansen IC, Corder RM, Nicolete VC, et al. Low-level *Plasmodium vivax* exposure, maternal antibodies, and anemia in early childhood: Population-based birth cohort study in Amazonian Brazil. PLoS Negl Trop Dis. 2021;15:e0009568. doi: 10.1371/journal.pntd.0009568.
