# Supplementary material for: SARS-CoV-2 seropositivity and COVID-19 among 5 years-old Amazonian children and their association with poverty and food insecurity
Source: PLoS Negl Trop Dis. 2022 Jul 18;16(7):e0010580. doi: 10.1371/journal.pntd.0010580 (PMC9292121; doi:10.1371/journal.pntd.0010580)
Supplement: S2 Fig — (PDF) [file pntd.0010580.s003.pdf]

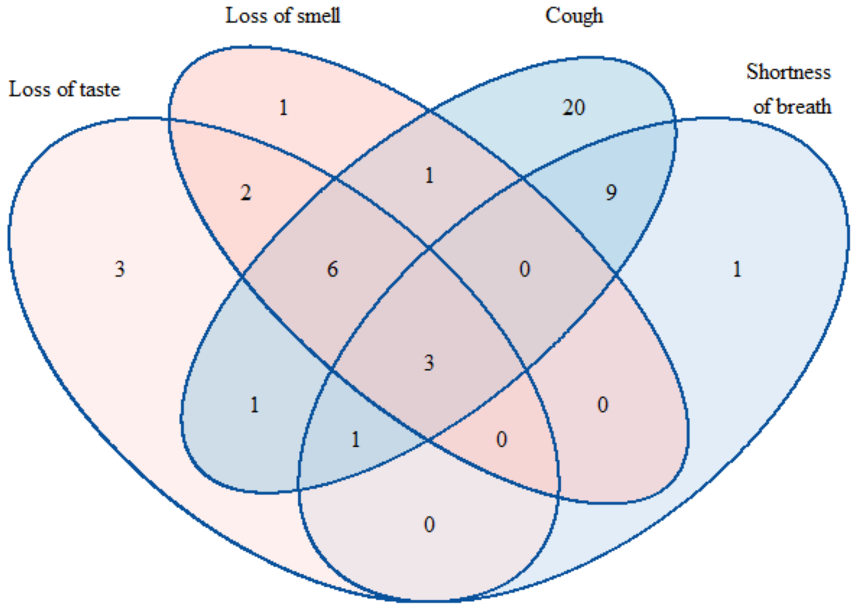

S2. Fig. Venn diagram of the four considered signs/symptoms experienced by SARS-CoV-2 seropositive children since the pandemic onset, as reported by mothers and guardians.
