# Supplementary material for: SARS-CoV-2 seropositivity and COVID-19 among 5 years-old Amazonian children and their association with poverty and food insecurity
Source: PLoS Negl Trop Dis. 2022 Jul 18;16(7):e0010580. doi: 10.1371/journal.pntd.0010580 (PMC9292121; doi:10.1371/journal.pntd.0010580)
Supplement: S1 File — (DOCX) [file pntd.0010580.s004.docx]

**S1 File: Supplementary Methods**

**Study design and population**

The Maternal and Child Health and Nutrition in Acre, Brazil (MINA-Brazil) study is a prospective, population-based birth cohort set-up in 2015 to examine the impact of a wide range of early exposures on child growth and development in the Amazon [1]. Mother-baby pairs were enrolled at pregnancy in public antenatal clinics, or at birth in the Women and Children’s Hospital of Juruá Valley, the only maternity hospital of Cruzeiro do Sul, where 96% of all local deliveries take place [2]. The study site is situated in the westernmost corner of Acre State, next to the Brazil-Peru border (S1 Fig). With 89,072 inhabitants estimated in 2020 by the Brazilian Institute of Geography and Statistics (IBGE) (https://www.ibge.gov.br/cidades-e-estados/ac/cruzeiro-do-sul.html), the municipality of Cruzeiro do Sul has 72% of its population classified as urban. The local infant mortality has been estimated in 2017 at 13.8 deaths among children under one year of age per 1,000 live births (https://www.ibge.gov.br/cidades-e-estados/ac/cruzeiro-do-sul.html), similar to the country estimate of 13.8 deaths per 1,000 live births ([https://biblioteca.ibge.gov.br/visualizacao/periodicos/3097/tcmb_2017.pdf](https://biblioteca.ibge.gov.br/visualizacao/periodicos/3097/tcmb_2017.pdf)).

**Baseline assessment**

Between July 2015 and June 2016, there were 1753 live births in the Women and Children’s Hospital of Juruá Valley [1]. At delivery, all mothers were invited to participate in the study; 18 (1.0%) did not complete the interview to obtain sociodemographic and morbidity information and 184 (10.5%) refused to participate. Therefore, 1551 mothers (88.5% of those eligible) were enrolled at the baseline. A total of 305 children (17.4%) whose mothers were interviewed at the baseline were not eligible for follow-up because they lived in remote rural areas, with difficult access to the city of Cruzeiro do Sul, where the 1-year, 2-years, and 5-years study follow-up visits were scheduled. The MINA-Brazil birth cohort population comprises 1246 children living in the urban area of Cruzeiro do Sul and nearby rural settlements – 71.1% of 1753 children born in the municipality of Cruzeiro do Sul during the enrolment period [1].

Sociodemographic information collected at the baseline included the duration of mother′s schooling (≤ 9, 10-12, >12 years), self-reported mother′s skin color (White [12%] vs. non-White [88%], the latter group comprising mothers self-identified as Brown [80%]; Yellow [4%], Black [3%], and Amerindian [1%]), mother′s occupation (unpaid vs. paid job), and whether the family is currently supported by the *Bolsa Família* conditional cash transfer program [3] (yes vs. no), a proxy of poverty. Data on selected household assets, collected at delivery and updated during the 5-year follow-up visit, were combined to derive a wealth index [4] as a proxy of socioeconomic status. Information on gestational age at delivery, and birth weight was retrieved from hospital records [1]. Information on breastfeeding practices (exclusive and total), recent morbidity (malaria and pneumonia since birth and over the past year as reported by mothers or guardians), and children′s growth and development was collected at 6-8 months, 1 year, 2 years, and 5 years of age during follow-up visits at a health center.

**Follow-up visit at 5 years of age**

Mothers were invited to bring their children to the scheduled 5-years follow-up assessment by using several communication strategies. Mothers who could not be reached by telephone calls, WhatsApp or Facebook messages were visited by trained health workers at the address reported at delivery to schedule the follow-up visit. Participant’s family or their relatives and friends were contacted if the mother could not be located. Of 1240 eligible children, 695 (56.0%) attended the 5-years follow-up assessment; there were 31 (2.5%) refusals and 514 (41.5%) losses (Fig 1, main text).

Child weight and height at 5 years of age were measured in duplicate. Body weight was measured with a digital electronic scale with 150 kg capacity and 100 g precision (UM061; Tanita Corporation, Arlington Heights, IL, USA). To measure height, we used a portable stadiometer (Alturaexata, Belo Horizonte, Brazil) with precision of 0.1 mm and an extension of 213 cm. Participants were barefoot and wore light clothes during measurements. Their nutritional status was classified based on critical z-score cut-off values with the Anthro software (https://www.who.int/childgrowth/en/), which uses the World Health Organization standard curves [5]. We considered the two most common conditions, stunting (stature-to-age: <-2 z-scores) and overweight (body mass index [BMI]-to-age: >2 z-scores). Wasting (BMI-to-stature: <-2 z-scores) was relatively rare in this population (1.3% prevalence at 5 years) and not considered in this analysis.

There was no COVID-19-related mortality in the study population. There was a single death among MINA Brazil cohort participants between the ages of 2 and 5 years (Fig 1, main text), but this event was not associated with COVID-19.

**Selection of covariates for the final Poisson regression models**

The effects of distal determinants, such as sociodemographic factors, on the risk of SARS-CoV-2 infection and COVID-19 are hypothesized not to be direct, but mediated by more proximate determinants, such as occupational, behavioral, and environmental factors [6]. For example, the effect of poverty on SARS-CoV-2 infection risk is mediated by more proximate factors such as poverty-related comorbidities (e.g., malnutrition), reduced access to healthcare, low-quality and overcrowded housing, and parents′ exposure to high-risk occupations [7]. Therefore, instead of introducing all variables at once in the multivariable model, under the implicit assumption that there is no causal hierarchy among them, and applying strictly statistical criteria to retain some of them in the final models, we took a hierarchical analysis approach for covariate selection. Further examples of conceptual frameworks applied to social and biological determinants of other diseases may be found in the recent reviews by Solar and Irvin [8] and Levene and colleagues [9].

Each covariate is allocated to one of three groups or “levels” (distal, intermediate, and proximal). The first level comprises the most important structural determinants of health: income, education, occupation, social class, gender, and race/ethnicity, while the next levels together comprise “intermediary” factors associated with the outcome [8]. In our analysis, covariates were allocated to the following groups: (1) distal level: child’s age and sex, mother′s skin color, household wealth index, mother′s schooling, mother′s occupation, whether the household is beneficiary of the *Bolsa Família* conditional cash transfer program, and household food insecurity; (2) intermediate level: perinatal factors (birth weight, gestational age, prematurity, breastfeeding); or (3) proximate level: nutritional status and morbidities at 5-year follow-up visit (anthropometric indicators, anemia, malaria).

We carried out an unadjusted analysis of the association between each covariate and the outcomes. Those associated with each outcome at *P* ≤0.20 was initially selected for multiple regression models. Afterwards, variable selection was done in a stepwise manner, starting with the most distal ones (sociodemographic characteristics). Within each level, covariates were retained in the subsequent analysis, until the most proximate level, if they were associated with the outcome at a significance level of <10% or if their inclusion in the model changed the risk measures by ≥10%.

**References:**

1. Cardoso MA, Matijasevich A, Malta MB, Lourenco BH, Gimeno SGA, Ferreira MU, Castro MC; MINA-Brazil Study Group. Cohort profile: the Maternal and Child Health and Nutrition in Acre, Brazil, birth cohort study (MINA-Brazil). BMJ Open. 2020;10:e034513. doi: 10.1136/bmjopen-2019-034513.

2. DATASUS, 2018. Sistema de Informação Sobre Nascidos Vivos, Cruzeiro do Sul, AC [in Portuguese]. http://www2.datasus.gov.br/DATASUS/index.php?area=060702.

3. Neves JA, Vasconcelos FAG, Machado ML, Recine E, Garcia GS, Medeiros MAT. The Brazilian cash transfer program (Bolsa Família): A tool for reducing inequalities and achieving social rights in Brazil. Glob Public Health. 2020:1-17. doi: 10.1080/17441692.2020.1850828.

4. Filmer D, Pritchett LH. Estimating wealth effects without expenditure data—or tears: an application to educational enrollments in states of India. Demography. 2001; 38: 115-132. doi: 10.1353/ dem.2001.0003.

5. World Health Organization. WHO child growth standards: length/height-for-age, weight-for-age, weight-for-length, weight-for-height and body mass index-forage: methods and development. Geneva; 2006. Accessed 8 February, 2022. Available at: https://www.who.int/childgrowth/standards/Technical_report.pdf

6. Victora CG, Huttly SR, Fuchs SC, Olinto MT. The role of conceptual frameworks in epidemiological analysis: a hierarchical approach. Int J Epidemiol. 1997;26:224-7. doi: 10.1093/ije/26.1.224.

7. Parolin Z, Lee EK. The role of poverty and racial discrimination in exacerbating the health consequences of COVID-19. Lancet Reg Health Am. 2022;7:100178. doi: 10.1016/j.lana.2021.100178.

8. Solar O, Irwin A. A conceptual framework for action on the social determinants of health. Social Determinants of Health Discussion Paper 2 (Policy and Practice). Geneva, World Health Organization, 2010. Available at: http://apps.who.int/iris/bitstream/handle/10665/44489/9789241500852_eng.pdf;jsessionid=D4C92CFEC9D3A36439EF78458D6C8F09?sequence=1

9. Levene LS, Bankart J, Walker N, Wilson A, Baker R. How health care may modify the effects of illness determinants on population outcomes: the Leicester SEARCH conceptual framework for primary care. BJGP Open. 2018;2:bjgpopen18X101603. doi: 10.3399/bjgpopen18X101603.
