# Supplementary material for: SARS-CoV-2 seropositivity and COVID-19 among 5 years-old Amazonian children and their association with poverty and food insecurity
Source: PLoS Negl Trop Dis. 2022 Jul 18;16(7):e0010580. doi: 10.1371/journal.pntd.0010580 (PMC9292121; doi:10.1371/journal.pntd.0010580)
Supplement: S1 Table — (DOCX) [file pntd.0010580.s005.docx]

**S1 Table.** Crude prevalence ratios (PR) and 95% confidence intervals (95% CI) for predictors of respiratory symptoms suggestive of respiratory infection (cough, shortness of breath, and/or loss of taste or smell in SARS-CoV-2-seronegative 5-year-old Amazonian children, as estimated by mixed-effects multiple Poisson regression models (n=363).

|  | PR | 95% CI | *P* |
| --- | --- | --- | --- |
| Child’s age (months) | 1.06 | 0.87 – 1.29 | 0.591 |
| Mother′s skin color*  White  Non-white | 1  3.17 | 0.42 – 24.11 | 0.264 |
| Household wealth index  1 (poorest)  2  3 (wealthiest)  *P* for trend | 1  0.46  0.60  0.341 | 0.33 – 0.62  0.20 – 1.81 | <0.001  0.367 |
| Mother′s schooling*  ≤9 years  10 to 12 years  >12 years  *P* for trend | 1  0.74  0.69  0.068 | 0.42 – 1.31  0.58 – 0.84 | 0.303  <0.001 |
| Household food insecurity  No  Yes | 1  0.92 | 0.62 – 1.38 | 0.687 |

*Missing values: mother’s skin color, n = 9; mother’s schooling, n = 10.
