# Supplementary material for: SARS-CoV-2 seropositivity and COVID-19 among 5 years-old Amazonian children and their association with poverty and food insecurity
Source: PLoS Negl Trop Dis. 2022 Jul 18;16(7):e0010580. doi: 10.1371/journal.pntd.0010580 (PMC9292121; doi:10.1371/journal.pntd.0010580)
Supplement: Attachment — Submitted filename: Rebuttal 3.docx [file pntd.0010580.s008.docx]

We once again thank the reviewers for their comments. We have now addressed the latest comments by reviewer #3:

1. Please note that the new Table S1 presenting predictors of symptoms suggestive of respiratory infection in the SARS-CoV-2 negative children show significant associations between the socioeconomic factors "household wealth index being beyond 1 (the poorest)", and "mothers schooling being beyond 9 years" and a significantly decreased risk of symptoms suggestive of respiratory infection. Thus, the concept socioeconomic status certainly predicts respiratory symptoms in the children under study, and this prediction is not SARS-CoV-2 specific. Although in the analysis of the subgroup of SARS-CoV-2 positive children, the associations were significant for other socioeconomical factors, namely "non-white mothers" and "food insecurity".

And residual confounding is - of course - present in the present observational study and must be acknowledged.

Added.

Fourth paragraph, Results:

“We found evidence that the socioeconomic status (children in the intermediate, but not the highest tertile, compared with the first tertile of wealth index) and >12 years of mother′s schooling were predictors of reduced risk of COVID-19-unrelated respiratory symptoms, with no clear dose-response relationship (S1 Table).”

Last paragraph, Discussion:

“We acknowledge respiratory symptoms caused by diseases other than COVID-19, but also associated with poverty (S1 Table), as a potential source of residual confounding in the association between clinically apparent COVID-19 and socioeconomic status. However, only 5.2% of the SARS-CoV-2 seronegative had COVID-19-like symptoms (cough, shortness of breath, and/or loss of taste or smell) during the study period and their prevalence was not significantly associated with mother′s skin color of household food insecurity (S1 Table).”

2. Significant associations were found between socioeconomic factors and respiratory symptoms, no matter if the children were SARS-CoV-2 positive or negative. This means that for example covid19-vacicination is not the cure. Improved socio-economy is a much better cure! I find that message of utmost importance. The point of socio-economy predicting transmission and severity of a broad range of infections, in the present study defined as respiratory symptoms, is indeed important and should be elaborated upon in the discussion.

We added:

Discussion section, third paragraph:

“Notably, study children from the poorest families are at increased risk of many other poverty-related childhood infections in addition to COVID-19 – including malaria, dengue, and intestinal and respiratory infections by other viruses, bacteria, and parasites – that may also severely affect their growth and development.”

Discussion, fourth paragraph:

“Strategies to mitigate poverty and food insecurity are urgently needed in the context of the current COVID-19 pandemic [36,40].”
