# Supplementary material for: SARS-CoV-2 seropositivity and COVID-19 among 5 years-old Amazonian children and their association with poverty and food insecurity
Source: PLoS Negl Trop Dis. 2022 Jul 18;16(7):e0010580. doi: 10.1371/journal.pntd.0010580 (PMC9292121; doi:10.1371/journal.pntd.0010580)
Supplement: Attachment — Submitted filename: Reviewer comments.docx [file pntd.0010580.s006.docx]

PNTD-D-22-00236: "SARS-CoV-2 infection and COVID-19 among 5 years-old Amazonian children and their association with poverty and food insecurity"

We appreciate the reviewers’ constructive comments and suggestions (here reproduced in blue) and believe that the revised manuscript should clarify all the concerns raised and be a lot clearer to readers.

Here we provide a detailed response to each comment and suggestion.

**Reviewer 1:**

Overall, it appears authors have an ongoing birth cohort from which they are using to assess the SARS-CoV-2 antibodies in children in Amazonian Brazil. This type of study is needed to fill the current gaps in the COVID literature, but I do recommend some revisions before full manuscript acceptance.

1. Currently as the manuscript stands there is a lot of information about the birth cohort and variable acquisition that may potentially benefit from paraphrasing and including these methods in a supplementary file for individuals who are more interested in the cohort conception. Authors should primarily focus on the methods specific to this analysis of prevalence of SARS-CoV-2 antibodies among children from Brazil, this way it is more clear and concise for readers.

Done. We moved the detailed description of the MINA Brazil birth cohort to the supplementary file S1 (Supplementary Methods). However, this section (now as part of the online supplement) was somewhat expanded to address some of the concerns raised by reviewer 2 (see below).

Methods:

2. Data Analysis: Authors decided to use Poisson regression to identify factors associated with: 1 SARS-CoV-2 infection and 2 COVID-19 with clinical symptoms and serological positive test. Authors state that they created a separate category for missingness within covariates. This type of approach is not ideal, I suggest authors revise their analysis to include multiple imputation of the missing data for the regression models.

Thank you for the comment. To address this point, we included the number of missing values for mother’s schooling (n= 15) and skin color (n=14) in Table 2, corresponding to 2.3% and 2.1%, respectively, of the total participants included in multiple models (n=660). There were no other missing values in the multiple models. Taking into account that our missing values occurred in very low frequencies and probably not at random, we have been using missing-value category based on literature recommendations (Sperrin, M., Martin, G.P. Multiple imputation with missing indicators as proxies for unmeasured variables: simulation study. *BMC Med Res Methodol* **20,** 185 (2020). <https://doi.org/10.1186/s12874-020-01068-x>).

.

3. Data Analysis: Authors use an alpha level of <0.20 to select variables to adjust for in the model. This approach is typically not ideal and leads to residual confounding. Ideally authors should use another methods such as change in estimate or directed acyclic graphs, unless authors are trying to truly identify "predictors". This is not clear in the methods section of the true goal of the multivariable model. Throughout the results it appears authors were trying to build more of a causal model. This lack of clarity has made my enthusiasm for the paper decrease.

We realize that our strategy for selecting covariates for the final model was poorly described in the original version. We added a new section to the Supplementary Methods online (S1 File) that describes in greater detail how we have selected variables for multivariable models.

As we explain in the main text (third paragraph, Data Analysis):

“Variables associated with the outcome at a significance level <20% in unadjusted analysis were entered in multiple Poisson regression models.”

Please note that the 20% significance level was used only to select the variables to be included in the initial Poisson regression models.

Next, we describe (in S1 File) the strategy used to retain variables in the final Poisson models:

“**Selection of covariates for the final Poisson regression models**

The effects of distal determinants, such as sociodemographic factors, on the risk of SARS-CoV-2 infection and COVID-19, are hypothesized not to be direct, but mediated by more proximate determinants, such as occupational, behavioral, and environmental factors [6]. For example, the effect of poverty on SARS-CoV-2 infection risk is mediated by more proximate factors such as poverty-related comorbidities (e.g., malnutrition), reduced access to healthcare, low-quality and overcrowded housing, and parents′ exposure to high-risk occupations [7]. Therefore, instead of introducing all variables at once in the multivariable model, under the implicit assumption that there is no causal hierarchy among them, and applying strictly statistical criteria to retain some of them in the final models, we took a hierarchical analysis approach for covariate selection. We introduced covariates within each level (distal, intermediate, and proximal) of determination in a stepwise approach, starting with the most distal ones (sociodemographic characteristics). Within each level, covariates were retained if they were associated with the outcome at a significance level of <10% or if their inclusion in the model changed the risk measures by ≥10%.”

Please note that we applied more strict criteria for retaining variables in the final model (last sentence, above).

Using the hierarchical approach described by Cesar Victora and colleagues in their seminal 1997 paper (reference 25, main text) to select covariates in multiple regression models does not imply that we have developed causal models. It simply provides a way to account for the hierarchical levels of determination of a given outcome while identifying outcome predictors.

Moreover, the approach addresses the issue of collinearity and redundancy among variables within the same hierarchical level – only variables associated with the outcome at a significance level of <10%, when controlling for other variables in the same or above levels, are retained in downstream analyses.

Finally, the changed the main text (third paragraph, Data Analysis) to state clearly the study objectives:

“Separate multiple Poisson regression models were built to identify predictors associated with each of two binary outcomes: (1) SARS-CoV-2 infection (using seropositivity as a proxy) regardless of any clinical signs and symptoms, and (2) clinically apparent COVID-19 upon serologically documented SARS-CoV-2 infection.

4. Laboratory methods for serological evidence: Currently authors have only stated that the nucleocapsid antigen is what was detected, was any method to detect spike protein or RBD?

We carefully revised the “SARS-CoV-2 antibody measurement” section to clarify that we searched for total (IgG and IgM) antibodies to the nucleocapsid antigen of SARS-CoV-2. We did not detect the “nucleocapsid antigen” and we did not attempt to detect antibodies to other antigens.

This serological approach has at least two advantages over the detection of antibodies to the spike protein: (a) vaccinated children do not develop antibodies to the nucleocapsid antigen, except for the few ones that received the inactivated Sinovac-CoronaVac COVID-19 vaccine; and (b) as we state in the main text, “the Elecsys assay to detect anti-nucleocapsid antibodies shows good signal stability, with little decay over time and rare seroreversions following a natural infection, being ideally suited for serosurveillance”. Because the time elapsed since infection is widely variable among study participants, we needed a stable test with very few seroreversions.

Reference 21 provides a detailed comparative analysis of the diagnostic performance of Elecsys and other antibody-detection assays. The authors conclude that: “For serosurveillance studies, direct, double Ag-sandwich assays appear to be the best choice due to their stability; in particular, algorithms that include both S1- and nucleocapsid-based assays can help reduce the rate of false-positivity and discriminate between natural infection and vaccine-derived seroreactivity.”

5. Results:

The results of the multivariable model do not match the goal of the analysis plan, recommend authors address whether they are trying to identify a causal model or identify predictors.

We carefully revised the abstract and main text to ensure that our study objectives are clearly stated.

The revised abstract includes the following sentence: “To address this knowledge gap, we investigate the prevalence and predictors of SARS-CoV-2 infection (either symptomatic or not) and disease in 5 years-old Amazonian children.”

The third paragraph of the Data Analysis section now clearly states the objective of the multivariable analysis, as described above (item 3).

6. Figure/Table placeholders are currently placed in the methods section, recommend authors consider putting these placeholders near the results they pertain to.

Fixed.

7. Discussion:

Overall, nice discussion and public health relevance is addressed. However, I do a couple of concerns that authors should address in the limitations section:

Authors do not address the time between potential exposure and seroconversion. We know that individuals can wane in detection overtime and this is a bias/flaw of the study design. This needs to be addressed of how this would impact the effect estimates; along if its differential/non-differential misclassification; and the degree to which the effect estimates may be impacted.

Please see the response to item 4. The Elecsys assay was chosen specifically because, due to its double-antigen sandwich format, it allows for detecting antibodies several months after the primary infection, with minimal (if any) signal decay. We therefore assume that this risk of misclassification due to antibody response waning is minimal.

**Reviewer #2:**

Author’s efforts to conduct research work and publish this study entitled “SARS-CoV-2 infection and COVID-19 among 5 years-old Amazonian children and their association with poverty and food insecurity” are appreciable. However, there are many issues related to this study. Some of the comments are as follows.

1. selected study population from ongoing enrolled cohort cannot be equated with general population as the opportunities to interact with care provider, counselling and heath care seeking behaviour are likely to be different.

We realize that some key features of the cohort study were poorly described in the original version and we revised the text accordingly.

We added to a new supplementary file (S1 File, Supplementary Methods) a more detailed description of the study design and population, reproduced below:

“**Study design and population**

The Maternal and Child Health and Nutrition in Acre, Brazil (MINA-Brazil) study is a prospective, population-based birth cohort set-up in 2015 to examine the impact of a wide range of early exposures on child growth and development in the Amazon [1]. Mother-baby pairs were enrolled at pregnancy in public antenatal clinics, or at birth in the Women and Children’s Hospital of Juruá Valley, the only maternity hospital of Cruzeiro do Sul, where 96% of all local deliveries take place [2]. The study site is situated in the westernmost corner of Acre State, next to the Brazil-Peru border (S1 Fig). With 89,072 inhabitants estimated in 2020 by the Brazilian Institute of Geography and Statistics (IBGE) (https://www.ibge.gov.br/cidades-e-estados/ac/cruzeiro-do-sul.html), the municipality of Cruzeiro do Sul has 72% of its population classified as urban. The local infant mortality has been estimated in 2017 at 13.8 deaths among children under one year of age per 1,000 live births (https://www.ibge.gov.br/cidades-e-estados/ac/cruzeiro-do-sul.html), similar to the country estimate of 13.8 deaths per 1,000 live births ([https://biblioteca.ibge.gov.br/visualizacao/periodicos/3097/tcmb_2017.pdf](https://biblioteca.ibge.gov.br/visualizacao/periodicos/3097/tcmb_2017.pdf)).

**Baseline assessment**

Between July 2015 and June 2016, there were 1753 live births in the Women and Children’s Hospital of Juruá Valley [1]. At delivery, all mothers were invited to participate in the study; 18 (1.0%) did not complete the interview to obtain sociodemographic and morbidity information and 184 (10.5%) refused to participate. Therefore, 1551 mothers (88.5% of those eligible) were enrolled at the baseline. A total of 305 children (17.4%) whose mothers were interviewed at the baseline were not eligible for follow-up because they lived in remote rural areas, with difficult access to the city of Cruzeiro do Sul, where the 1-year, 2-years, and 5-years study follow-up visits were scheduled. The MINA-Brazil birth cohort population comprises 1246 children living in the urban area of Cruzeiro do Sul and nearby rural settlements – 71.1% of 1753 children born in the municipality of Cruzeiro do Sul during the enrolment period [1].

Sociodemographic information collected at the baseline included the duration of mother′s schooling (≤ 9, 10-12, >12 years), self-reported mother′s skin color (White [12%] vs. non-White [88%], the latter group comprising mothers self-identified as Brown [80%]; Yellow [4%], Black [3%], and Amerindian [1%]), mother′s occupation (unpaid vs. paid job), and whether the family is currently supported by the *Bolsa Família* conditional cash transfer program [3] (yes vs. no), a proxy of poverty. Data on selected household assets, collected at delivery and updated during the 5-year follow-up visit, were combined to derive a wealth index [4] as a proxy of socioeconomic status. Information on gestational age at delivery, and birth weight was retrieved from hospital records [1]. Information on breastfeeding practices (exclusive and total), recent morbidity (malaria and pneumonia since birth and over the past year as reported by mothers or guardians), and children′s growth and development was collected at 6-8 months, 1 year, 2 years, and 5 years of age during follow-up visits at a health center.

**Follow-up visit at 5 years of age**

Mothers were invited to bring their children to the scheduled 5-years follow-up assessment by using several communication strategies. Mothers who could not be reached by telephone calls, WhatsApp or Facebook messages were visited by trained health workers at the address reported at delivery to schedule the follow-up visit. Participant’s family or their relatives and friends were contacted if the mother could not be located. Of 1240 eligible children, 695 (56.0%) attended the 5-years follow-up assessment; there were 31 (2.5%) refusals and 514 (41.5%) losses (Fig 1, main text). “

Here we highlight a few important aspects:

(a) First, all mothers delivering between July 2015 and June 2016 in the only maternity hospital of Cruzeiro do Sul were invited to participate in the baseline evaluation. Of 1753 eligible mothers, 1551 (88.5%) were enrolled. Next, 305 children living in remote rural areas (17.4% of those enrolled) were not eligible for the follow-up assessment. Therefore, the MINA Brazil cohort comprises 1246 children – the vast majority of those born between July 2015 and June 2016 and living in the urban area of Cruzeiro do Sul and nearby rural communities.

(b) We minimized the effect of variation in health seeking behavior by scheduling study assessments with our research team (including medical doctors, nurses, nutritionists, and technicians) separately from routine child care visits and by using several strategies (telephone calls, WhatsApp or Facebook messages, and home visits) to ensure participation in the these assessments.

2. Inclusion of a control group from general population was desirable.

Thanks for raising this point.

We showed above that the MINA Brazil cohort has enrolled the vast majority of children born between July 2015 and June 2016 and living in the urban area of Cruzeiro do Sul and nearby rural communities. There were 1240 children eligible for the 5-years follow-up assessment and 695 (56.0%) attended it. This means that roughly half of the 5 years-old children living in the city and nearby rural communities were interviewed and tested for SARS-CoV-2 antibodies.

Who are left to serve as “general population controls” in this age group? The remaining 545 children in this age group who originally entered the MINA Brazil cohort and lived in the study area? These children were either lost to follow-up, because they have moved from the study area or could not be located (n = 514), or refused to participate in the assessment (n= 31; see Fig 1, main text). These, of course, are not suitable population controls. We argue that the 5 years-old children living in Cruzeiro do Sul that would be available for antibody testing were actually examined in the present study.

Importantly, we highlight in the main text (third paragraph of the “Study design and population” section) that:

“Children participating in the 5-year assessment and those lost to follow-up (n = 514) had similar perinatal characteristics regarding sex, gestational age, preterm birth, and birth weight, but differed significantly in the proportion of children from poorest families (36.2% vs. 44.1%) and of mothers with ≤9 years of schooling (26.4% vs. 47.5%), respectively, with *P* <0.01 (χ^2^ test) for both.”

Therefore, the (already high) prevalence of SARS-CoV-2 antibodies detected in the study population may underestimate the seroprevalence of COVID-19 in the general population. In other words, a more extensive sampling of the target age group (including more children from the poorest families) would potentially reveal an even greater spread of COVID-19 in young children.

We acknowledge this study limitation in the last paragraph of “Discussion” section:

“Children lost to follow-up differ from those participating in the present serosurvey in key correlates of socioeconomic vulnerability, such as socioeconomic status and mother′s education, suggesting that SARS-CoV-2 infection and disease could be even more common in the original study population.”

3. This data pertains to the pandemic survivors only. There is no information about those children who lost their life due to covid or related complications.

We added the following sentence to the Supplementary Methods (S1 file, last paragraph of the “Follow-up visit at 5 years of age” section):

“There was no COVID-19-related mortality in the study population. There was a single death among MINA Brazil cohort participants between the ages of 2 and 5 years (Fig 1, main text), but this event was not associated with COVID-19.”

4. Based on the reports from other countries, most vulnerable children for covid complications and adverse outcome are in the preschool age group (especially infants) or adolescence. Rationale for screening at 5 years of age is not clear.

Children younger than 5 or 6 years (depending on which vaccine is applied, whether Sinovac-CoronaVac [rarely used in this age group] or Pfizer) are the only age group that remains ineligible for COVID-19 vaccination in Brazil. Information regarding the prevalence of infection and disease remains scarce in this age group, especially because young children (wrongly, as we show here) perceived to be at low risk of infection. Accordingly, we show that the prevalence of infection is grossly underestimated in young children in the Amazon:

“Here we show that 45% of 660 participants in a birth cohort study in the Brazilian Amazon had SARS-CoV-2 antibodies at the age of 5 years, although only 5% of them reported previously diagnosed COVID-19 episodes” (Author summary).

Importantly, the relative role of young children in spreading SARS-CoV-2 – in populations where older children, adolescents, and adults are vaccinated – remains little investigated. We changed the main text to highlight that:

“Young children, who are not routinely vaccinated, appear to be more likely to transmit SARS-CoV-2 to household contacts once infected, compared with older children and adolescents [8]”.

As the reviewer correctly state, children at the age of 5 years appeared to be less vulnerable to COVID-19 complications than older children and adults in the studies carried out in affluent societies. However, how socioeconomic inequalities may affect the risk of disease, upon SARS-CoV-2 infection, among young children living in the tropics remains undetermined. The present study suggests that poverty and food insecurity may indeed increase the risk of clinically apparent COVID-19 in this age group.

5. Basic population characteristics with regard to immunisation status, anemia, chronic infections and other comorbidities are not addressed

As suggested, we added this information in Table 1.

6. Title of the study is not representative of work done and need modifications with regard to Covid sero-positivity

Fixed. The title was changed to: "SARS-CoV-2 seropositivity and COVID-19 among 5 years-old Amazonian children and their association with poverty and food insecurity"

7. Conclusions are not supported by the study and represent more of general statement.

We revised the main text to make sure that the main conclusions of the study are clearly stated.

(a) Abstract: “Childhood SARS-CoV-2 infection and COVID-19-associated illness are substantially underdiagnosed and underreported in the Amazon. Children in the most socioeconomically vulnerable households are disproportionately affected by SARS-CoV-2 infection and disease.”

(b) Main text: We added a new section, “Conclusion”, after “Discussion”. It reads:

“ The present study provides new insights into the epidemiology of SARS-CoV-2 infection and COVID-19 and its association with social inequalities in young Amazonian children. We show that SARS-CoV-2 infections are frequent but substantially underreported among 5 years-old children in the Brazilian Amazon. Children in the most socioeconomically vulnerable households are disproportionately affected by SARS-CoV-2 infection. Those living in households experiencing food insecurity and born to non-White mothers are more likely to have COVID-19-related morbidity once infected.”

We highlight some very specific conclusions of the study, rather than more general observations. First, we showed that COVID-19 was grossly underreported in the study population: “only 15 (5.1%; 95% CI, 2.9-8.2%) of the seropositive children had a prior medical diagnosis of COVID-19 reported by their mothers or guardians” (first paragraph, “High SARS-CoV-2 seroprevalence but low COVID-19-related morbidity”).

We also showed that the risk of SARS-CoV-2 seropositivity and disease is positively associated with poverty: “Children from the poorest households and those with less-educated mothers remained significantly more likely to be seropositive at the age of 5 years after controlling for potential confounders (Table 2). Similarly, children from households experiencing food insecurity and those born to non-White mothers were more likely to have had COVID-19-related morbidity by the age of 5 years, after adjustment for confounders (Table 2).” (“Higher risk of SARS-CoV-2 infection and COVID-19 among socioeconomically vulnerable children” section).

8. Author have listed many limitations of this study, substantiating a fact that it may not be possible to draw a meaningful conclusion from this study and possibly does not add much to existing knowledge.

Reassuringly, reviewer #1’s opinion about the Discussion was as follows: “Overall, nice discussion and public health relevance is addressed.” We acknowledge that the manuscript must describe the study conclusions and their public health significance in a way that readers with different interests and backgrounds can readily identify the main study’s contributions to the literature. We have extensively revised the manuscript to make this point clearer.
