# Supplementary material for: SARS-CoV-2 seropositivity and COVID-19 among 5 years-old Amazonian children and their association with poverty and food insecurity
Source: PLoS Negl Trop Dis. 2022 Jul 18;16(7):e0010580. doi: 10.1371/journal.pntd.0010580 (PMC9292121; doi:10.1371/journal.pntd.0010580)
Supplement: Attachment — Submitted filename: Reviewer comments 2.docx [file pntd.0010580.s007.docx]

PNTD-D-22-00236: "SARS-CoV-2 infection and COVID-19 among 5 years-old Amazonian children and their association with poverty and food insecurity"

We appreciate the reviewers’ constructive comments and suggestions, here highlighted in blue. We have revised the manuscript in order to address all concerns raised. We numbered all comments and suggestions and here we provide a detailed response to each of them.

**Reviewer #1:**

1. Throughout the text, some sentences appear in a lighter text and different font. Recommend revising.

Fixed.

2. From my prior concern: "Authors do not address the time between potential exposure and seroconversion. We know that individuals can wane in detection overtime and this is a bias/flaw of the study design. This needs to be addressed of how this would impact the effect estimates; along if its differential/non-differential misclassification; and the degree to which the effect estimates may be impacted."

It is recommended authors add a summarized version of their response/rationale to this comment in the limitations section of the manuscript. Ensure you state regardless of the method used the type of misclassification bias and the degree this may impact your effect estimates.

Done. Please see the last paragraph of the Discussion section:

“Second, antibody measurements were carried out during two separate study rounds, with substantial SARS-CoV-2 transmission occurring between them (Fig 2A). Moreover, the time elapsed between potential exposure and detection of seroconversion varies widely among study participants and seroprevalence rates are not directly comparable between surveys. We used two approaches to cope with this limitation. We used a serological test with stable signal over time and rare seroreversions following a natural infection [21] to minimize the risk of differential misclassification (participants infected during the first pandemic wave might be more likely to yield a false-negative serology due to waning antibody responses). In addition, we used mixed-effects regression models to account for the clustering of participants within study rounds, as participants in each round are expected to differ in their previous exposure to (and time elapsed since) SARS-CoV-2 infection.”

**Reviewer #3:**

Reviewer #3: Thank you for letting me review this interesting and relevant study.

Major revisions are needed to Methods, Results and Discussion.

1. The retrospective study design is a limitation.

We added a brief discussion of this limitation to the last paragraph of the Discussion section.

2. It is not clear how the study was planned and carried out. Is the design overall explorative? Specifically, was there any pre-plan of outcomes, or based on what were they selected? If the outcome selection was based on preliminary results, the quality (i.e. replicability) of the results will be low because of high risk of Type 1 error. In the same line of thought, were there any pre-planned statistical analysis plan?

Following the suggestions by reviewers #1 and #2, the description of the original cohort study was considerably shortened and details are given in the Supplementary Methods (S1 File). The cohort study was set up in 2015 “to examine the impact of a wide range of early exposures on child growth and development”. A detailed account of the cohort set-up is provided in reference 12.

Of course, COVID-19 was not included among the exposures and outcomes at the time the MINA cohort study was designed. However, given the dramatic COVID-19 crisis across the Amazon in the early 2020 (first wave) and 2021 (second wave), estimating the attack rate of SARS-CoV-2 infection in our cohort population and its clinical consequences emerged as a priority in the context of participants’ follow-up.

This is an exploratory study (as we now emphasize in the last paragraph of Introduction). At the time we decided to measure SARS-CoV-2 antibody levels in our cohort participants, we had no prespecified hypothesis regarding predictors of infection and disease in addition to those described in the literature – including poor housing, food insecurity and other poverty-related conditions.

No preliminary results were available for selecting/defining outcomes. One of them (past SARS-CoV-2 infection) is straightforward. The second outcome (past clinically apparent COVID-19) was defined based on selected reported symptoms in the same way as vaccine efficacy endpoints were defined in COVID-19 vaccine trials. Similar approaches were used in our previous COVID-19 serosurveys (e.g., reference 20).

3. It is essential to address whether the findings are SARS-CoV-2/COVID19-specific, or in fact pertinent to airway infections in general: The analyses must include an (exploratory) analyses with another composite outcome of any cough, shortness of breath, loss of taste or smell (and no regard to +/- SARS-CoV-2 results). Such approach will test if (the same? or other?) socioeconomic factors determine symptoms often experienced by individuals having airway infections in general. Using symptoms of gastrointestinal infection as the explorative composite outcome, the same approach would test if (the same? or other?) socioeconomic factors determine infections in general. Such input is of outmost importance to the overall message: If socioeconomic factors determine any infection (which is likely), specific vaccination programs will not suffice when it comes to securing better health globally.

We use a highly specific assay to examine the association between SARS-CoV-2 antibody seropositivity and socioeconomic factors. Given the high specificity of the assay (estimated at 99.8% in reference 24), the risk of misclassification (children with other respiratory diseases being misclassified as having experienced SARS-CoV-2 infection) is minimal.

Regarding the first study outcome (SARS-CoV-2 seropositivity). Of course, our children may have experienced other respiratory infections during the study period, some of them known to be associated with poverty, but these other infections would not change their SARS-CoV-2 antibody status. Not surprisingly, some risk factors for SARS-CoV-2 seropositivity may be also associated with increased risk of other poverty-related infections: diarrhea, intestinal parasitic infections, bacterial pneumonia. We do not argue that poverty is exclusively associated with SARS-CoV-2. Specifically answering the question whether “using symptoms of gastrointestinal infection as the explorative composite outcome, the same approach would test if (the same? or other?) socioeconomic factors determine infections in general?”, the most likely answer is yes, but we did not obtain detailed information regarding laboratory-confirmed intestinal infections during the follow-up of the MINA cohort participants. We added a comment on this topic to the end of the third paragraph of the Discussion section.

Now we address the second study outcome (SARS-CoV-2 seropositivity and symptoms). Symptoms associated with other infections might theoretically inflate the proportion of SARS-CoV-2 infections that we classified as “clinically apparent” or “symptomatic” COVID-19. We added the following (underlined) to the third paragraph of the Discussion section:

“The COVID-19 pandemic has exacerbated pre-existing socioeconomic and health disparities worldwide [30] which, in turn, increase the risk of SARS-CoV-2 infection and COVID-19 morbidity in children. This is due to poverty-related comorbidities (e.g., malnutrition), reduced access to healthcare, low-quality and overcrowded housing, and parents′ exposure to high-risk occupations [31]. We note, however, that some SARS-CoV-2 seropositive children may have been misclassified as having experienced clinically apparent COVID-19 in our study if they had other poverty-related respiratory infections leading to cough, shortness of breath, or loss of taste or smell.”

We also discuss the (limited) sensitivity and specificity of our symptom list in the last paragraph of the Discussion section:

“Third, the symptoms investigated retrospectively are not necessarily specific for childhood COVID-19 and may be affected by recall bias. Some symptoms may have been caused by other locally prevalent infections experienced by their children since the COVID-19 pandemic started, such as malaria. Only the unadjusted analysis showed that the 17 children with self-reported malaria within the past 12 months were more likely to report one or more clinical signs and symptoms associated with COVID-19 (Table 1), suggesting that our definition of clinically apparent COVID-19 may have been, at least in part, confounded by malaria-associated symptoms. Fourth, to minimize the risk of false positives, we excluded from our symptom/sign list some clinical manifestations that, although not specific, are commonly reported in childhood COVID-19. These include fever, vomiting, diarrhea, and headache [22]. For example, seropositive children with reported fever but without cough, shortness of breath, and loss of taste or smell have not been classified as having experienced clinically apparent COVID-19. Moreover, symptom reports are prone to recall bias and mild clinical manifestations may have been overlooked by mothers and guardians. Consequently, we surely have missed some clinically apparent COVID-19 cases in our study population.”

However,

“Among the 363 seronegative children, only 19 (5.2%; 95% CI, 3.2-8.1%) had a report of cough, shortness of breath, and/or loss of taste or smell during the study period.” (Second paragraph of the Results section).

These data suggest that cough, shortness of breath, and/or loss of taste or smell associated with respiratory infections other than COVID-19 are unlikely to have confounded our analysis.

In fact,

“The presence of these same clinical symptoms (cough, shortness of breath, and/or loss of taste or smell) was not significantly associated with household food insecurity or mother′s skin color among SARS-CoV-2 seronegative children (n = 363; S1 Table).” (Fourth paragraph of the Results section).

We added a new supplementary table (S1 Table) with these results.

4. Based on what was predictors selected? Also after reading the authors' answer to prior reviewer comments, the processes of predictor selection and statistical modelling are not clearly described and argued for. For each round of analyses (crude, adjusted, final adjusted), please provide information on assumptions, criteria and evidence base, inclusive considerations about each factor (for example is household food insecurity considered a socioeconomic factor, or a somatic factor (hunger and malnutrition leading to less efficient organ function and immune responses)?

Following the suggestions by reviewers #1 and #2, part of the statistical analysis section was moved from the main text to the Supplementary Methods section online (S1 File).

As requested, we added to S1 File the list of covariates considered in each level of analysis. Hopefully, this additional information will help the reader to follow the process of variable selection using the hierarchical framework. We decided not to provide a very detailed description of the hierarchical framework because (a) it falls beyond the scope of the present study and (b) because it has been very extensively used in epidemiologic research since its original formulation in the 1997 article by Victora and colleagues, which received 1314 citations in the literature (Web of Science as of early May, 2022). However, we provide in S1 File an additional reference that discusses the use of the hierarchical framework in broader health-related contexts, where the interested reader may find a lengthy discussion on the assumptions and application of the analytical approach. The revised text makes it clear that household food insecurity was considered as a distal sociodemographic factor.

In S1 File, we provide a description of the criteria used to retain covariates in the final models:

“The effects of distal determinants, such as sociodemographic factors, on the risk of SARS-CoV-2 infection and COVID-19 are hypothesized not to be direct, but mediated by more proximate determinants, such as occupational, behavioral, and environmental factors [6]. For example, the effect of poverty on SARS-CoV-2 infection risk is mediated by more proximate factors such as poverty-related comorbidities (e.g., malnutrition), reduced access to healthcare, low-quality and overcrowded housing, and parents′ exposure to high-risk occupations [7]. Therefore, instead of introducing all variables at once in the multivariable model, under the implicit assumption that there is no causal hierarchy among them, and applying strictly statistical criteria to retain some of them in the final models, we took a hierarchical analysis approach for covariate selection. Further examples of conceptual frameworks applied to social and biological determinants of other diseases may be found in the recent reviews by Solar and Irvin [8] and Levene and colleagues [9].

Each covariate is allocated to one of three groups or “levels” (distal, intermediate, and proximal). The first level comprises the most important structural determinants of health: income, education, occupation, social class, gender, and race/ethnicity, while the next levels together comprise “intermediary” factors associated with the outcome [8]. In our analysis, covariates were allocated to the following groups: (1) distal level: child’s age and sex, mother′s skin color, household wealth index, mother′s schooling, mother′s occupation, whether the household is beneficiary of the *Bolsa Família* conditional cash transfer program, and household food insecurity; (2) intermediate level: perinatal factors (birth weight, gestational age, prematurity, breastfeeding); or (3) proximate level: nutritional status and morbidities at 5-year follow-up visit (anthropometric indicators, anemia, malaria).

We carried out an unadjusted analysis of the association between each covariate and the outcome. Those associated with each outcome at *P* ≤0.20 was initially selected for multiple regression models. Afterwards, variable selection was done in a stepwise manner, starting with the most distal ones (sociodemographic characteristics). Within each level, covariates were retained in the subsequent analysis, until the most proximate level, if they were associated with the outcome at a significance level of <10% or if their inclusion in the model changed the risk measures by ≥10%. “

5. Figure 2: Information on deaths in the overall population is irrelevant and may be misleading. Please delete information on mortality from the figure.

Done.

6. Figure 3: How can the percentages be the exact same in the two analyses?

The percentages indicate the proportions of contacts who reported one of more previous COVID-19 episodes. The first analysis (upper panel) focuses on risk of being seropositive among children (outcome: detection of anti-SARS-CoV-2 antibodies), while the second (lower panel) focuses on the risk of having COVID-19 (outcome: detection of anti-SARS-CoV-2 antibodies plus report of clinical symptoms). Note that the denominators (numbers of children at risk) are the same in both analyses; only the numerators differ. Therefore, the proportions of children exposed to contacts reporting previous COVID-19 episodes are exactly the same in both analyses.

We added an explanation to Figure 3 legend.

7. Any considerations about multiplicity?

What is meant by “multiplicity” in this context is unclear to us. Does the reviewer refer to the multiple comparison problem, in which correlates of different independent outcomes are tested in parallel? The current study examines only two outcomes and they are not independent of each other – i.e., we are not carrying out two independent comparisons. As explained above (response to item 6) and in the main text (Materials and Methods, Outcome Definitions), our outcomes are: (a) SARS-CoV-2 seropositivity and (b) SARS-CoV-2 seropositivity plus reported symptoms. Therefore, a study participant will not be classified as having experienced COVID-19 (outcome 2) if s/he is not seropositive (outcome 1). We argue that the multiple comparison problem does not apply to our analysis of just 2 interdependent outcomes.

8. Line 265 - 267: Why "interestingly"? It is well known - also for SARS-CoV-2 - that crowding, especially indoor crowding, increases the risk of transmission. Please note, as mentioned above, that transmission patterns cannot be inferred from the present study where no information on index case is available (please see Discussion below).

We deleted the word “interestingly”.

9. Lines 290 - 293 the authors conclude that their data point towards small children being a source of SARS-CoV-2-household transmission. That cannot be concluded based on the present study, since data on the timing of infection (i.e. index cases) were not available. It may be most likely that the SARS-CoV-2 is introduced to the family by the adolescents or adults who have more contacts outside the family.

We changed the text to highlight that our cross-sectional analysis does not allow for inferring the direction of household transmission (whether from young children to older household members or the other way around):

“We acknowledge that our cross-sectional analysis does not allow us to infer the direction of SARS-CoV-2 transmission between children and their close contacts. However, literature data suggest that adults are more likely than children to acquire COVID-19 from a household or family index case [26]. We hypothesize that asymptomatic young children [8], especially those with relatively high viral loads [6,7], may be a significant source of household transmission to adults in settings characterized by poor housing conditions, little compliance with social distancing, and limited availability of diagnostic testing to identify subclinical SARS-CoV-2 carriage. Whether this applies to our study participants remains to be investigated by considering (currently unavailable) information on time of infection of each household member.” (Second paragraph of the Discussion section).
